# An Open-Label Study of a Wearable Device Targeting ADHD, Executive Function, and Academic Performance

**DOI:** 10.3390/brainsci13121728

**Published:** 2023-12-18

**Authors:** Lindsay E. Ayearst, Richard Brancaccio, Margaret Danielle Weiss

**Affiliations:** 1 Revibe Technologies, 3331 Heritage Trade Dr, Suite 101, Wake Forest, NC 27587, USA; rich.brancaccio@revibetech.com; 2 Cambridge Health Alliance, Harvard University, Boston, MA 02459, USA; madweiss@cha.harvard.edu

**Keywords:** ADHD, wearables, attention, executive function

## Abstract

Objective: This was an open-label pilot study to test the feasibility and preliminary efficacy of a wearable digital intervention developed to improve on-task behavior. This was an exploratory study to test for specificity of response on parent- and teacher-reported symptom outcomes in attention and hyperactive/impulsive symptoms, as well as domains of functional impairment, including school behavior and learning and executive function. Method:Participants included 38 children aged 8–12 years with a parent-reported past diagnosis of ADHD. Following baseline ratings from parents (*N* = 38) and teachers (*N* = 26), participants wore the device to school for four weeks. Parent and teacher ratings of ADHD symptoms, executive function, and functional impairment were repeated at the end of the four-week intervention period. Results:Statistically significant improvement was seen in the total scores for all parent and nearly all teacher outcomes, with moderate effect size improvements in attention, organization and planning, self-monitoring, school functioning, and teacher-reported academic performance. Conclusions: Preliminary evidence from this open-label pilot study suggests that having a child interact with a wearable device to self-monitor attention is feasible. This exploratory, open-label pilot study found real-world improvement in functional domains, including academic performance. Future research will require a blinded, randomized, controlled trial using an appropriate sham comparator to confirm these findings.

## 1. Introduction

The DSM-5 diagnosis of Attention-Deficit/Hyperactivity Disorder (ADHD) is based on difficulty in either or both of two domains: attention and hyperactivity/impulsivity. Symptoms of hyperactivity and impulsivity tend to be more readily observable and reported by virtue of the overt behavioral disruption these symptoms may cause, where external observation of difficulty with attention is more challenging [1,2]. Individuals with ADHD may ‘look’ like they are paying attention when, in actuality, their mind has wandered, or, alternatively, they may ‘look’ like they are off task because they are moving around when, in fact, they are processing what is going on. As a result, individuals with ADHD who primarily experience attention problems, such as girls, patients with “Cognitive Disengagement Syndrome” (previously known as “Sluggish Cognitive Tempo”; [3,4]), or older adults (>55 years) [5,6] tend to be underdiagnosed.

Multiple investigations have demonstrated that it is attention deficit that is most persistent longitudinally [7,8], strongly associated with difficulty in executive function [9,10], and may be inclusive of symptoms that are the most influential in producing or maintaining the disorder [11,12]. Using a novel network analysis, Martel and colleagues [12] argued that the central symptoms of ADHD from preschool to young adulthood are “difficulties sustaining attention” and being “easily distracted”, suggesting that they may warrant extra weighting in future diagnostic systems. They viewed these two symptoms as key treatment targets, as changes to these symptoms likely impact the ADHD network as a whole. Multiple studies have demonstrated that attention selectively impacts academic performance [13,14], which is, in turn, critical to education and, ultimately, vocational success.

Psychological treatments for ADHD, such as parent training, are by and large directed towards behavior management [15]. There is some evidence to suggest that psychological interventions that specifically target attention symptoms administered in the classroom can be effective [16,17,18]. This may include attention deficit, as it occurs either in the context of the Combined or the Inattentive presentations. A variety of digital devices have been developed more recently that have attempted to train attention using computer games. The first such intervention was Cogmed, which seemed promising initially but failed to show far-transfer effects to real-life outcomes in randomized controlled trials (RCTs) [19]. More recently, Akili Interactive completed several studies demonstrating the effect of a computerized game on outcomes as measured by one subtest of a computerized evaluation of ADHD, the Test of Variables of Attention (TOVA; [20,21]). However, the computer game did not show effectiveness for ADHD symptoms overall or functional impairment compared to control [22]. One might hypothesize that the failure to show far-transfer effects may, in part, be because the practice is limited to the home setting.

Barkley and others have pointed out that for any intervention to be effective, it must occur at the point of performance [23,24]. If this were the case, the effectiveness of attention training by a digital device would need to occur in the classroom to improve on-task behavior at school. The device would have to be able to learn when the child is paying attention, as well as the variables that impact attention, and provide continuous but non-stigmatizing feedback to reinforce attention. No psychological or digital intervention to date has been able to achieve this.

Revibe Connect is a wearable watch-like device that incorporates unique vibration signals that leverage principles of meta-cognitive awareness and self-monitoring to improve attention. Self-monitoring is one of the most common behavioral interventions for improving deficits in self-regulation, with over 50 years of research supporting its efficacy for a range of diverse learners, including youth with ADHD (see [25] for a more complete description of the evidence supporting self-monitoring as a behavioral intervention for improving on-task behavior). The device vibrates intermittently and asks the child to “tap back” to indicate whether they are paying attention. The device was developed to target attention and reduce off-task behavior by combining the advantages of three recent technological innovations: wearable devices sensitive to physiological parameters, algorithmically-guided adaptive response, and machine-learning-driven digital assessment and feedback. The device learns the child’s needs over time and in real-time, providing a personalized, adaptive, and unobtrusive intervention at the point of performance.

Therapeutically, the device serves several different functions. By transferring “pay attention” reminders from a person to a device, the Revibe Connect increases on-task behavior while minimizing the cost and burden of stigmatizing teacher or paraprofessional support in the classroom. The device operates continuously with high frequency in the moment and in situ in the classroom, increasing the potential to train attention in the classroom setting. The device is continuously collecting data to measure attention, thus creating an opportunity for caregivers and teachers to become more aware of the environmental determinants of attention symptoms. For example, the device may reveal the child shows better attention to one teacher over another, with a particular subject, or at a particular time of day. The device also provides data-driven personalized behavioral insights based on real-world user activity, such as “when Mary takes 3000 steps in the morning, her focus rate in the afternoon is improved by 12%.” This information can then be used to restructure the environment to optimize attention-promoting circumstances. The device also provides a tool for recording in-the-moment self-reported attention, which may complement rating scales, computerized tests, or neuropsychological evaluation.

The current pilot study aimed to investigate near-transfer effects on ADHD symptoms and far-transfer effects on executive function, teacher-reported academic performance, and functional impairment in the context of an open-label investigation of youth with ADHD aged 8–12 years over four weeks who were utilizing Revibe Connect.

## 2. Materials & Methods

### 2.1. Subjects

Participants were recruited via online advertisements and social media directed at parents of children with an existing diagnosis of ADHD. Inclusion criteria were the willingness of the teacher to participate and access to a mobile device that could download the Revibe Connect application. Teachers were required to (1) have known the youth for at least four weeks and (2) be providing in-person classroom learning to the youth during the study period (as opposed to virtual or hybrid learning). Youth participants had to: (1) have a diagnosis of ADHD from a health care professional, (2) be between 8–12 years of age, and (3) not be receiving any pharmacological treatment for ADHD during the study and in the period 30 days prior to enrolment in the study. Exclusion criteria for youth included (1) having a parent-reported physical or motor condition that would prevent tapping the device to provide self-reported on/off-task behavior when prompted or result in the child being unable to feel the vibrations from the device on their wrist and (2) having ever worn or used Revibe Connect prior to the study. Data was collected between July and December 2021. All parents and teachers provided informed consent, and youth participants provided assent. This study was reviewed and approved by WCG IRB on 7 June 2021 (IRB Tracking #:20212935).

### 2.2. Measures

All measures were completed via an online link, with the instruction that the measures be completed independently by each rater, in one sitting and in a quiet environment. Measures were collected at baseline and again following the completion of the four-week intervention. Given the exploratory nature of this study, a wide selection of measures of both symptoms and functioning were included to help inform a future planned RCT.

#### 2.2.1. ADHD

ADHD symptoms were assessed using the ADHD Rating Scale-5 (ADHD-RS-5, [26]), normed for children aged 5 to 17 years, using parent- and teacher-specific norms for attention, hyperactive-impulsive symptoms, and total score. Higher scores are indicative of greater severity of symptoms. Ratings are based on behavior observed over the previous six months or since the beginning of the school year. For the purposes of this research, the time period of six months was changed to the past four weeks to reflect on the period of time the child was wearing the device. ADHD outcome was also assessed with the parent and teacher short versions of the Conners 4 Short Form [27], which, in addition to measuring the core symptoms of ADHD, includes evaluation of executive function, emotional dysregulation, and impairment across functional domains, such as schoolwork, peer interactions, and family life. Higher scores are indicative of greater difficulties. Ratings are based on the past month, which is consistent with the time period of the intervention.

#### 2.2.2. Executive Function

Executive function was measured with the Comprehensive Executive Function Inventory (CEFI; [28]) parent and teacher forms. The CEFI provides a total score and nine domain scales. For scoring purposes, the raw scores are converted to standard scores with a mean of 100 and a standard deviation of 15, with higher scores indicating better executive function. Ratings are based on the past four weeks, which is consistent with the intervention period of the current study.

#### 2.2.3. Functional Impairment

Domain-specific parent observation of functional impairment secondary to ADHD symptoms was evaluated with the Weiss Functional Impairment Rating Scale—Parent From (WFIRS-P; [29]). The WFIRS-P consists of 50 items and collects the parent’s perspective of their child’s functioning in the family, school learning and behavior, life skills, self-concept, and social and risky activities. Higher scores are indicative of greater impairment. Ratings are based on the past month. The WFIRS-P has been widely used in clinical trials, as it is sensitive to change with treatment.

To assess teacher observations of impairment and to obtain a comparison between parent and teacher ratings of impairment, we also included the Impairment Rating Scale (IRS; [30]). The IRS is a brief, generic parent and teacher rating scale for assessing impairment in youth with ADHD. The parent version has seven domains (relationship with peers, relationship with siblings, relationship with parents, academic progress, self-esteem, influence on family functioning, and overall impairment). The teacher version has six domains (relationship with peers, relationship with teachers, academic progress, self-esteem, influence on classroom functioning, and overall impairment). Ratings were made on a seven-point Likert scale, as suggested in Fabiano et al., 2006 [30], with higher scores indicative of greater impairment.

#### 2.2.4. Academic Performance

To demonstrate real-life effectiveness in remediating the deficits in academic performance associated with ADHD, teachers completed the Academic Performance Rating Scale (APRS; [31]). This is a 19-item scale that was developed to reflect teachers’ perceptions of students’ academic performance and abilities in classroom settings. It includes items directed towards work performance in various subject areas (e.g., “Estimate the percentage of written math work completed relative to classmates”), academic success (e.g., “What is the quality of this child’s reading skills?”), behavioral control in academic situations (e.g., “How often does the child begin written work prior to understanding the directions?”), and attention to assignments (e.g., “How often is the child able to pay attention without you prompting him/her?”). The items are rated on a 5-point Likert scale and summed, resulting in a total score and three subscales: Academic Success (scores range from 7–35), Impulse Control (scores range from 3–15), and Academic Productivity (scores range from 12–60). Higher scores indicate greater classroom academic performance over the past week.

### 2.3. Procedures

Eligible parents invited the teacher to participate. Parents completed an online consent and attested that their child had assented, at which point the baseline measures were sent via an online link, and once completed, the wearable device was mailed to the family. The parents were instructed that the child must wear the device during school hours but could continue to wear the device at home as well if desired (e.g., during homework time). Parents received nightly notifications in the app to remind them to charge and sync the device. Compliance with wearing the device was monitored remotely by Revibe staff on a daily basis.

On the last day of the four-week intervention period, emails were sent to the parent and teacher with a link to complete the post-treatment questionnaire. Teachers were compensated for their participation with a gift card, and parents were allowed to keep the device and given their choice of a gift card or to continue using Revibe with a free one-year membership.

### 2.4. Digital Treatment

The Revibe Connect uses customized, patented vibration signals to redirect the user to on-task behavior and collects and records self-monitored data. The user responds to the vibration cues by physically tapping the device once if they are off task or twice if they are on task. The device records the behavior, as well as gyroscope and accelerometer data, and outputs a variety of metrics, including attention span (estimation of the longest possible interval that the child was on task, based on self-reported data), focus rate (self-reported on-task behavior, percentage of “yes” responses divided by total responses), response rate (percentage of time the user responded to vibration prompts, total responses divided by total prompts), AI-driven step counting, estimated caloric burn, and minutes spent fidgeting (AI-driven metric). The device personalizes the frequency of the vibration signals, based on both real-time and historical feedback provided by each user, while also operating according to pre-determined floor and ceiling levels (to avoid being a distraction and to ensure the child does not go too long without a reminder). The vibration signals to the wrist of the user remind them to focus and get back on task. The vibration cues vary in sensation to avoid habituation to the signal (patent-protected, anti-habituation technology: US 10,624,590 B2 Issued April 21, 2020; EP 3 010 573 B1 Issued 14 April 2020), and the strength of the vibration can be set to low, moderate, or strong, based on the sensitivity needs of the child. Text reminders tailored to the child’s needs and schedule can be entered (by caregivers via the app) to appear on the screen at pre-set times to help bolster executive function (e.g., “Don’t forget your homework”). Finally, a combination of both traditional and deep neural network analyses are used to create personalized insights for each user (for example, “When Johnny takes 2000 or more steps between 8 a.m. and 12 p.m., focus rate for the rest of the day is 30% higher”). Notably, these insights were not provided to parents via the app in the current study, as insights are based on having a minimum of 20 days of valid data (where valid means removing days that are outliers) within a 90-day period. Based on the parameters of the study, no one would have reached the minimum threshold to receive a data-driven insight.

Overall, the user experience is quite straightforward. The device sends a vibration to the child’s wrist, and when the child feels the vibration, they are to ask themselves, “Am I doing what I’m supposed to be doing?”. The child then responds to the device by tapping once if they are off task or twice if they are on task. The device acknowledges the child’s response by providing a “tap back” that matches the response given by the child. The frequency of the vibrations is determined by considering the recent series of responses to the vibration, as well as using historical feedback data reported for that same time of day. As a result, for example, a child may receive more prompts during math class, where they tend to have trouble staying on task, and fewer prompts during language arts, where they typically report greater rates of on-task behavior. The frequency of the vibration cues is, therefore, tailored to each child, with the average number of vibration cues ranging from 50 to 60 prompts per day (based on data from a large customer database accumulated over time from when the device was available for purchase). Using the app, parents/caregivers are asked to schedule times when they want the child to receive prompts (e.g., classroom instructional time) and not include non-instructional times, such as recess and lunch, when on-task behavior need not be monitored.

### 2.5. Statistical Analysis

All analyses were based on the change between the means of pre- and post-intervention scores using a two-tailed paired *t*-test and associated Cohen’s *d* effect size measurement. A Shapiro–Wilk test for normality was performed for all scales. If the distribution of scores was found to deviate from normality at an alpha of less than 0.05, an equivalent Wilcoxon signed-rank test was performed instead of a *t*-test, and the associated rank-sum correlation effect size was estimated. Due to the exploratory nature of these analyses, all results less than an alpha of 0.05 were considered statistically significant. For completeness, the tables also report if the alpha levels met significance under a Bonferroni correction within a statistical family to control for Type 1 error rates. All analyses were conducted using SPSS version 29.0 [32].

Additional post-hoc analyses were conducted to determine the percent of patients who would be considered treatment responders based on the minimal clinically important difference (MCID; [33]) for each scale. ADHD symptom response was defined as a 30% or more reduction in symptoms based on the ADHD-RS-5 [34]. For standardized scales such as the Conners 4 and CEFI, we followed the rule of thumb that a 0.5 SD change is a good approximation of MCID [35]. Functional response was defined as a decrease in WFIRS-P total score of ≥0.25 [36] and an improvement of ≥1 point on the IRS [22]. In addition to the responder analyses, post-hoc analyses were conducted to determine whether self-reported focus rate and attention span collected using the device (tap-back feedback from the youth) aligned with the parent- and teacher-reported symptom change.

## 3. Results

### 3.1. Device Compliance

This study took place from July to December 2021, during the pandemic when children were often absent from school. Other difficulties with compliance included challenges with ensuring the device had been charged, synced, and was on the child’s wrist before they left for school.

Devices were shipped to a total of 80 participants; 77 of those participants set up their devices. Of those, 73 of them wore the device at least once, with an average of 14 active days, and 7% of those participants wore the device for all 20 days (four weeks, Monday to Friday). An additional five subjects were excluded for the following reasons: lost to follow-up (n = 1, not completing the post-intervention questionnaire, this participant also only wore the device for 1 day), the child lost the device (n = 2, the device was lost after the fourth and eighth day of use), the parent withdrew from the study (n = 2, parent reported their child was bothered by the vibration sensation after six days of usage, and the other withdrew after four days of use, noting dissatisfaction with the device due to trouble syncing). This left 68 participants eligible for inclusion in the analyses.

Subjects were considered to have sufficient exposure to the device if they used it for a minimum of three out of five days per week or a minimum of 15 days out of 20 over the course of the intervention period, which avoided penalizing those who had sufficient exposure but were impacted by COVID-19. For example, a child who had missed an entire week of school due to COVID-19 but had worn it every other day (15/20 days) would have greater exposure to the device than a child who wore it a minimum of three out of five days per week (for a total of 12/20 days). The criterion was also data-driven, based on data analysis from our large database of users, where change is seen almost immediately and sustained (based on as little as two days exposure per week sustained over three weeks) [37]. Based on this dual criterion, the sample consisted of 43 children (63% of the total) with an average of 17 out of 20 days of active use (range 13–20 days). While parents were encouraged to have their child wear the device to school, they were not limited to use during school hours. The device could be worn after school and in the evening (e.g., while doing homework). Thirty-eight percent of participants entered schedules that went beyond the school day. On average, participants had six hours per day (range 3.2 to 12.4) of classes or activities scheduled in the application for personalized vibrations to be delivered to the child’s wrist. The average number of vibration prompts per child per day was 62 (SD = 23). The response rate was consistent over the trial period. There was no statistically significant difference between the response rate at baseline (64%) versus week 1 through week 4, suggesting that there was no evidence of habituation to the vibration prompt over time.

### 3.2. Participants

Of the 43 cases eligible for inclusion in the analyses, five cases were excluded because of protocol violations (taking medication during the study period, hybrid learning, or absence of an existing ADHD diagnosis). Two teachers were removed from the analysis because they had known the child for less than four weeks. The final sample consisted of ratings from 38 parents and 26 teachers. Demographic characteristics of the participants are reported in Table 1.

### 3.3. Symptom Change

Significant improvement was reported by parents on the ADHD-RS-5 over the treatment period, with a large effect size (Cohen’s *d* ≥ 0.80) on symptoms of inattention (*d* = 1.07) and a moderate effect size (Cohen’s *d* between 0.50–0.79) on symptoms of hyperactivity/impulsivity (*d* = 0.70) (Table 2). Teachers reported significant improvement in the ADHD-RS-5 attention domain, with a moderate effect size (*d* = 0.54), but an improvement in symptoms of hyperactivity/impulsivity was not statistically significant (Table 3). Fifty-eight percent of children were responders based on parent-reported improvement on the ADHD-RS total score, and 35% were based on teacher reports (indicated in the “% Responders” column of Table 2 and Table 3).

Responses for parent and teacher reports on the Conners Short Form 4 are reported in Table 2 and Table 3, respectively. Large improvements were noted with parent reports for inattention/executive dysfunction, hyperactivity, impulsivity, and schoolwork. Teachers reported a moderate effect size improvement on inattention/executive dysfunction and schoolwork.

Mean change in focus rate and attention span across weeks was evaluated to determine if the device metrics, based on the child’s in-the-moment self-reported behavior, aligned with the results reported by the parents and teachers, with respect to large (parent) to moderate (teacher) effect size changes in attention. Due to the distribution of scores not meeting the assumptions of normality, a Wilcoxon signed-rank test was performed, and the associated Z statistic was reported. Effect sizes are interpreted as small (<0.30), moderate (0.30 to 0.49), and large (≥0.50). Table 4 and Table 5 indicate that consistent with the parent- and teacher-reported observed changes in attention, self-reported focus rate showed a statistically significant improvement over baseline with moderate (week 1) to large (weeks 2–4) effect sizes. Similarly, attention span showed statistically significant improvement over baseline, with a moderate effect size in weeks 2–4.

### 3.4. Executive Function

Significant improvement was noted in the parent evaluation for the full-scale score of the CEFI and for each of the nine domain scales, with a large effect size on all the domain scales except for flexibility, where a moderate effect was noted (Table 6). The effect size of teacher-rated executive function on the CEFI was large for self-monitoring and moderate for the other domains, with the exception of emotional regulation and inhibitory control (Table 7). Seventy-nine percent of children met the criteria for being responders on the CEFI based on the Full-Scale score, as reported by parents, and 35% as reported by teachers.

### 3.5. Functional Impairment and Academic Performance

A statistically significant reduction in impairment was noted on the IRS total score, both by parents and teachers, with a moderate effect size (Table 8 and Table 9). Parents noted a significant reduction in the overall severity of impairment and better academic progress, with large effect sizes on both dimensions. Moderate effect sizes were observed in terms of a reduction in impairment in self-esteem, relationship with parent(s), and impact on the family, as well as the total score (average rating across the six domains). With the teacher’s evaluation, moderate effects were noted on reduction in impairment in terms of peer and teacher relationships, impact on the classroom, and academic progress, as well as the total score. Post-hoc analyses examining responses to the intervention showed that 37% of children were responders, according to parents, and 35% were responders, according to teachers.

A significant improvement was noted in school learning, with a large effect size on the WFIRS-P (Table 10) and a moderate effect size improvement in school behavior and life skills. A post-hoc responder analysis indicated that 37% of participants demonstrated a ≥0.25 reduction in total functioning score on the WFIRS.

Similarly, significant improvement in academic productivity was noted by teachers on the APRS, with a moderate effect size (Table 11).

## 4. Discussion

The findings of this open-label pilot study provide preliminary support for the utility of this wearable digital intervention to improve ADHD symptoms, executive function, overall functioning, and academic performance in children with ADHD.

The Revibe Connect was designed to specifically target improvement in attention and on-task behavior. The most notable finding in this pilot study is evidence to support that the device is meeting that objective. The finding that effects were most robust for attention symptoms versus hyperactive and impulsive symptoms, parent-reported school learning versus behavior, and teacher-reported academic performance suggests that the device may represent a specific, psychological, non-medication option to improve attention. Teacher-rated improvements and treatment response rates suggest that the device is working at the point of performance in the classroom. Given that in previous research, the collateral report has been more sensitive to behavior than attention [38,39], evidence of these within-trial differences is promising as a signal that the device may have a specific impact on attention. Although this was an open-label study with anticipated placebo effects, we would assume that placebo effects would be equally distributed across the various outcomes. This is the first point-of-performance digital intervention to demonstrate both near-transfer effects on ADHD symptoms and far-transfer effects on real-life executive function, school, and academic performance.

While the device is designed as a point-of-performance intervention for use in both classroom and home settings, in the current study, the majority of participants wore the device only during school hours (38% had scheduled hours that went beyond the school day). Despite the focus of this study being on the classroom setting, it was the parents who reported observing the largest improvements and strongest effects. It is possible that this was, in part, driven by those using the device at home (e.g., during homework time after school, as well as text reminders for completing household chores or routine activities). This would result in fewer parent/child negative interactions, as the watch (instead of the parent) was doing the nagging. Further, parents were often in contact with teachers, either directly (with the teacher calling the parent to report an incident that occurred in class) or via daily or weekly report cards. Fewer calls home to the parent from the teacher, positive feedback in the log, or noticing that more work is being completed (e.g., more questions completed on tests) were all things that could lead to the parent reporting improvement, even though the parent was not in the school setting. Alternatively, the effects being reported by parents may represent a stronger placebo effect, as somewhat inflated improvements are not uncommon in parent-reported outcomes compared to those reported by teachers in medication trials [40].

The strengths of the study are: (1) the inclusion of well-validated outcome measures for ADHD symptoms, executive function, academic performance, and functional impairment (to help inform measure selection for a future large-scale pivotal trial), (2) the inclusion of both parent and teacher raters, and (3) the evaluation of both statistically significant improvement and treatment response (MCID). As an indication that parents were satisfied with the treatment, it is notable that over 50% of parents selected to continue to use the device with their child (a free one-year membership, valued at $199) over an Amazon gift card (valued at $100)) as compensation for their participation. While the value of a one-year membership was greater than the value of the gift card, the gift card provided instant gratification and could be used towards any Amazon purchase, whereas the membership only had value if parents believed the device was helping their child.

### Limitations

The study has significant limitations. The open-label design makes it difficult to determine the extent to which the observed improvement was, at least in part, a placebo effect, or could result from regression to the mean or the passage of time. Furthermore, on multiple measures, the overall severity of symptoms at baseline was modest, thus limiting the variance to show change or to determine how these results would replicate in a moderately ill clinical population. It is possible that the more modest baseline symptoms could be related to having recruited children who were unmedicated, which could skew the symptom severity of the sample downward. Having said this, 87% of the sample had a baseline ADHD-RS of 24, which is at the 88th percentile of boys aged 8–10 (93rd percentile for girls) and 89th percentile of boys aged 11–13 (94th percentile for girls). It should also be noted that symptoms were measured systematically by both parent and teacher, and all subjects had sufficient concern about difficulty with symptoms to motivate them to seek treatment with a device that was demanding of time and organization. The duration of the trial was short and may have led to a failure to show the true optimal response to the device over time. Further, results could be due to a novelty effect that may wane with time. Future studies will need to explore whether the effects endure over a longer period of time. The sample size was small, especially relative to the number of outcome variables; however, for an exploratory pilot study, the identification of sensitive outcomes and protocol parameters carried more importance for future research design than minimizing Type I error, although many findings remained significant after Bonferroni correction (as noted in the tables). While the study did find significant near and far transfer effects for the group, data on the percentage of treatment responders suggest that this treatment may be effective for a selective population. Evaluation of Cohen’s effect size using a repeated measures design is usually larger than what is reported for between-group differences in a RCT. Compliance was compromised by the pandemic, with students being sent home due to COVID-19 and having to remain at home until a negative COVID-19 test could be provided, and there were challenges getting ADHD families to consistently use a device.

## 5. Conclusions

This open-label pilot study provides preliminary evidence for the feasibility of utilizing a wearable device to enable children to self-monitor attention. The within-study findings of this pilot study support the device’s potential to selectively enhance attention and learning over behavior. This study represents the first empirical evaluation of a device designed to target attention, executive function, and academic performance at the point of performance. The Revibe Connect, serving as a non-medication strategy to improve attention, holds promising clinical implications, providing a novel therapeutic avenue for children with ADHD and executive function deficits. Future research will test this hypothesis with a sham-controlled, randomized clinical trial in a larger sample of children with ADHD as determined by structured diagnostic interviews.

## Figures and Tables

**Table 1:** Demographic Characteristics of Rated Youth.

Demographic		Parent		Teacher	
Age (in years)		***N***	**%**	***N***	**%**
	8	2	5.3	1	3.8
	9	8	21.1	6	23.1
	10	8	21.1	3	11.5
	11	10	26.3	9	34.6
	12	10	26.3	7	26.9
	M (SD)	10.5 (1.2)		10.5 (1.3)	
Gender	Boys	26	68.4	20	77
	Girls	12	31.6	6	23
U.S. Race/Ethnicity	% Hispanic	3	8	1	4
	Asian	1	3	1	3.8
	Black	4	11	2	7.7
	White	30	79	20	76.9
	Other	3	8	3	11.6
Parental Education Level	High school graduate/GED	1	2.6	0	0
	Some college or associate’s degree	9	23.7	8	31
	Bachelor’s degree or higher	28	73.7	18	69
ADHD Diagnosis	ADHD Inattentive	14	36.8	9	35
	ADHD Hyperactive/Impulsive	3	7.9	3	11
	ADHD Combined	18	47.4	13	50
	Don’t Know	3	7.9	1	4
Comorbidity	Anxiety	8	21.1	6	23.1
	Autism Spectrum Disorder	2	5.3	1	3.8
	Depression	2	5.3	2	7.7
	ODD	2	5.3	2	7.7
	Tic	1	2.6	1	3.8
	Specific Learning Disability	24	63.2	11	42.3
Total		38	100	26	100

**Table 2:** Assessment of improvement in symptoms based on parent evaluations.

	PARENT N = 38								
Scale	Mean-Pre (SD)	Mean-Post (SD)	Mean Difference (SD)	95% CI (L|U)	Test Statistic (T)	*p* Value	Effect Size	95% CI of ES (L|U)	% Responders
ADHD-RS-5									
Inattention	20.31 (4.8)	13.71 (6.3)	6.6 (6.2) **	4.6|8.6	6.603	<0.001	1.07	0.67|1.5	58%
Hyperactivity/Impulsivity	12.95 (6.3)	9.18 (5.8)	3.77 (5.3) **	2.0|5.5	4.336	<0.001	0.70	0.34|1.0	58%
ADHD Total Score	33.26 (8.5)	22.89 (10.3)	10.37 (10.3) **	7.0|13.7	6.204	<0.001	1.01	0.61|1.4	58%
CONNERS 4—SHORT									
Inattention/Executive Dysfunction	73.63 (6.7)	62.55 (9.4)	11.08 (9.1) **	8.1|14.1	7.458	<0.001	1.21	0.78|1.6	76%
Hyperactivity	67.5 (11.9)	59.34 (9.0)	8.16 (8.8) **	5.3|11.0	5.721	<0.001	0.93	0.54|1.3	63%
Impulsivity	65.63 (12.9)	57.08 (11.1)	8.55 (10.2) **	5.2|11.9	5.164	<0.001	0.84	0.46|1.2	61%
Emotional Dysregulation	63.79 (13.5)	58.95 (13.5)	4.84 (8.9) **	1.9|7.8	3.343	0.002	0.54	0.20|.88	47%
Schoolwork	70.42 (10.1)	59.66 (10.2)	10.76 (9.8) **	7.5|14.0	6.754	<0.001	1.10	0.69|1.5	71%
Peer Interactions	61.82 (14.0)	56.11 (13.2)	5.71 (11.0) **	2.1|9.3	3.201	0.003	0.52	0.18|.85	55%
Family	62.68 (13.3)	58.42 (14.3)	4.26 (10.8)	.71|7.8	2.429	0.02	0.39	0.06|.71	47%

** Indicates statistical significance after a Bonferroni correction of 0.05/3 = 0.017 (ADHD-RS-5) and 0.05/7 = 0.007 (Conners 4—Parent). For each variable, the normality assumption for *t*-tests was verified using a Shapiro–Wilks test. If the Shapiro–Wilks test indicated that the distribution of scores did not meet normality, a Wilcoxon signed-rank test was performed instead. In the “Test Statistic” column, this is indicated by a (T) or (Z) after the test statistic, indicating if a *t*-test (T) or a Wilcoxon test (Z) was performed. *p* values were calculated according to the statistical test run. Effect sizes are calculated as Cohen’s *d* when a *t*-test was performed and *r* if a Wilcoxon signed-rank test was performed. Effect Size interpretation: Cohen’s *d*—large ≥ 0.80, moderate 0.50–0.79, small 0.21–0.49, negligible ≤ 0.20; *r*—large ≥ 0.50, moderate 0.30–0.49, small < 0.30. “% Responders” represents the percentage of participants in the sample who met the MCID for improvement.

**Table 3:** Assessment of improvement in symptoms based on teacher evaluations.

	TEACHER N = 26								
Scale	Mean-Pre (SD)	Mean-Post (SD)	Mean Difference (SD)	95% CI (L|U)	Test Statistic (T/Z)	*p* Value	Effect Size	95% CI of ES (L|U)	% Responders
ADHD-RS-5									
Inattention	15.08 (8.0)	11.65 (7.3)	3.43 (6.3) **	0.85|6.0	2.746 (T)	0.011	0.54	0.12|0.95	38%
Hyperactivity/Impulsivity	7.81 (8.1)	6.19 (6.4)	1.62 (4.2)	−0.10|3.3	−1.72 (Z)	0.085	−0.34	−0.61|0.02	35%
ADHD Total Score	22.88 (14.0)	17.85 (12.1)	5.03 (8.3) **	1.7|8.4	−2.911 (Z)	0.004	−0.57	−0.80|0.25	35%
CONNERS 4—SHORT									
Inattention/Executive Dysfunction	64.19 (12.7)	58.62 (10.3)	5.57 (9.28) **	1.83|9.33	3.063 (T)	0.005	0.60	0.18|1.01	50%
Hyperactivity	55.27 (13.9)	53.23 (11.4)	2.04 (7.5)	−1.0|5.1	−1.369 (Z)	0.171	−0.27	−0.66|0.02	35%
Impulsivity	56.85 (11.8)	53.31 (8.3)	3.54 (7.94)	0.33|6.74	2.274 (T)	0.032	0.45	0.04|0.84	38%
Emotional Dysregulation	51.12 (10.5)	50.96 (10.7)	0.16 (6.8)	−2.6|2.9	−0.159 (Z)	0.874	−0.03	−0.44|−0.01	12%
Schoolwork	60.12 (12.8)	56.31 (10.6)	3.81(9.5)	−0.03|7.6	−2.025 (Z)	0.043	−0.40	−0.62|−0.02	31%
Peer Interactions	54.73 (12.8)	53.77 (8.9)	0.95 (10.8)	−3.4|5.3	−0.334 (Z)	0.739	−0.07	−0.43|−0.01	23%
Family	-	-	-	-	-	-	-	-	-

** Indicates statistical significance after a Bonferroni correction of 0.05/3 = 0.017 (ADHD-RS-5) and 0.05/6 = 0.008 (Conners 4—Teacher). For each variable, the normality assumption for *t*-tests was verified using a Shapiro–Wilks test. If the Shapiro–Wilks test indicated that the distribution of scores did not meet normality, a Wilcoxon signed-rank test was performed instead. In the “Test Statistic” column, this is indicated by a (T) or (Z) after the test statistic, indicating if a *t*-test (T) or a Wilcoxon test (Z) was performed. *p* values were calculated according to the statistical test run. Effect sizes are calculated as Cohen’s *d* when a *t*-test was performed and *r* if a Wilcoxon signed-rank test was performed. Effect Size interpretation: Cohen’s *d*—large ≥ 0.80, moderate 0.50–0.79, small 0.21–0.49, negligible ≤ 0.20; *r*—large ≥ 0.50, moderate 0.30–0.49, small < 0.30. “% Responders” represents the percentage of participants in the sample who met the MCID for improvement.

**Table 4:** Improvement in mean focus rate over baseline across the intervention period.

	Focus Rate % Mean (SD)	Mean Change from Baseline	% Increase over Baseline	Z	*p*	r
Baseline	48.37 (19.85)					
Week 1	56.11 (23.00)	7.74	16%	−2.64	0.008	−0.43
Week 2	62.00 (23.85)	13.63	28%	−3.456	<0.001	−0.56
Week 3	62.24 (25.61)	13.87	29%	−3.119	0.002	−0.51
Week 4	61.47 (26.77)	13.10	27%	−3.097	0.002	−0.50

**Table 5:** Improvement in mean attention span over baseline across the intervention period.

	Attention Span (Minutes) Mean (SD)	Mean Change from Baseline	% Increase over Baseline	Z	*p*	r
Baseline	10.45 (18.73)					
Week 1	10.34 (9.53)	−0.11	−1%	−1.798	0.072	−0.29
Week 2	13.21 (15.03)	2.76	26%	−2.557	0.011	−0.41
Week 3	15.16 (18.21)	4.71	45%	−2.745	0.006	−0.45
Week 4	14.24 (13.76)	3.79	36%	−2.488	0.013	−0.40

**Table 6:** Improvement in Domains of Executive Function by Parent Raters.

PARENT N = 38									
Scale	Mean-Pre (SD)	Mean-Post (SD)	Mean Difference (SD)	95% CI (L|U)	Test Statistic (T/Z)	*p* Value	Effect Size	95% CI of ES (L|U)	% Responders
CEFI									
Attention	74.76 (9.0)	88.71 (9.2)	−13.95 (9.4) **	−17|−10.9	−9.168 (T)	<0.001	−1.49	−1.9|−1.0	71%
Emotion Regulation	85.95 (16.9)	97 (14.1)	−11.05 (9.8) **	−14.3|−7.8	−4.892 (Z)	<0.001	−0.79	0.73|0.87	61%
Flexibility	82.03 (10.9)	88.39 (13.1)	−6.36 (10.8) **	−9.9|−2.8	−3.624 (T)	<0.001	−0.59	−0.93|−0.24	47%
Inhibitory Control	81.66 (12.1)	92.68 (11.8)	−11.02 (11.9) **	−14.9|−7.1	−4.659 (Z)	<0.001	−0.75	0.61|0.86	66%
Initiation	71.92 (11.4)	87.58 (13.5)	−15.66 (11.3) **	−19.4|−11.9	−8.556 (T)	<0.001	−1.39	−1.8|−0.94	87%
Organization	71.74 (8.7)	85.87 (10.4)	−14.13 (10.8) **	−17.7|−10.6	−8.06 (T)	<0.001	−1.31	−1.7|−0.87	79%
Planning	78.58 (9.8)	89.45 (11.2)	−10.87 (9.7) **	−14.1|−7.7	−6.884 (T)	<0.001	−1.12	−1.5|−0.71	68%
Self-Monitoring	73.42 (11.3)	88.66 (13.3)	−15.24 (14.5) **	−20.0|−10.5	−6.479 (T)	<0.001	−1.05	−1.4|−0.65	76%
Working Memory	68.74 (10.8)	87.76 (11.7)	−19.02 (12.2) **	−23.0|−15.0	−9.621 (T)	<0.001	−1.56	−2.0|−1.1	87%
Full Scale	73.79 (9.6)	88.39 (10.8)	−14.6 (10.8) **	−18.1|−11.1	−8.36 (T)	<0.001	−1.36	−1.8|−0.91	79%

** Indicates statistical significance after a Bonferroni correction of 0.05/10 = 0.005. For each variable, the normality assumption for *t*-tests was verified using a Shapiro–Wilks test. If the Shapiro–Wilks test indicated that the distribution of scores did not meet normality, a Wilcoxon signed-rank test was performed instead. In the “Test Statistic” column, this is indicated by a (T) or (Z) after the test statistic, indicating if a *t*-test (T) or a Wilcoxon test (Z) was performed. *p* values were calculated according to the statistical test run. Effect sizes are calculated as Cohen’s *d* when a *t*-test was performed and *r* if a Wilcoxon signed-rank test was performed. Effect Size interpretation: Cohen’s *d*—large ≥ 0.80, moderate 0.50–0.79, small 0.21–0.49, negligible ≤ 0.20; *r*—large ≥ 0.50, moderate 0.30–0.49, small < 0.30. “% Responders” represents the percentage of participants in the sample who met the MCID for improvement.

**Table 7:** Improvement in Domains of Executive Function by Teacher Raters.

Scale	Mean-Pre (SD)	Mean-Post (SD)	Mean Difference (SD)	95% CI (L|U)	Test Statistic (T)	*p* Value	Effect Size	95% CI of ES (L|U)	% Responders
CEFI									
Attention	82.08 (14.0)	89.81 (13.3)	−7.73 (10.7) **	−12.0|−3.4	−3.682	0.001	−0.72	−1.1|−0.28	42%
Emotion Regulation	97.31 (14.2)	101.58 (15.4)	−4.27 (13.4)	−9.7|1.1	−1.628	0.116	−0.32	−0.71|.08	38%
Flexibility	85.92 (8.7)	90.81 (9.7)	−4.89 (8.6)	−8.3|−1.4	−2.896	0.008	−0.57	−0.98|−0.15	38%
Inhibitory Control	90.96 (12.3)	95.19 (11.4)	−4.23 (11.3)	−8.8|0.34	−1.906	0.068	−0.37	−0.77|.03	38%
Initiation	79.46 (15.0)	86.04 (14.6)	−6.58 (10.7) **	−10.9|−2.3	−3.143	0.004	−0.62	−1.0|−0.19	42%
Organization	81.54 (11.9)	89.15 (12.9)	−7.61 (11.1) **	−12.1|−3.1	−3.483	0.002	−0.68	−1.1|−0.25	46%
Planning	84.23 (10.8)	91.31 (10.6)	−7.08 (9.0) **	−10.7|−3.4	−4.019	<0.001	−0.79	−1.2|−0.34	42%
Self-Monitoring	84.46 (11.1)	92.42 (10.5)	−7.96 (10.0) **	−12|−3.9	−4.057	<0.001	−0.80	−1.2|−0.35	46%
Working Memory	82.62 (14.4)	90.85 (12.5)	−8.23 (12.6) **	−13.3|−3.1	−3.317	0.003	−0.65	−1.1|−0.22	50%
Full Scale	83.81 (12.0)	91.04 (11.8)	−7.23 (10.0) **	−11.3|−3.2	−3.691	0.001	−0.72	−1.1|−0.28	35%

** Indicates statistical significance after a Bonferroni correction of 0.05/10 = 0.005. For each variable, the normality assumption for *t*-tests was verified using a Shapiro–Wilks test. If the Shapiro–Wilks test indicated that the distribution of scores did not meet normality, a Wilcoxon signed-rank test was performed instead. In the “Test Statistic” column, this is indicated by a (T) or (Z) after the test statistic, indicating if a *t*-test (T) or a Wilcoxon test (Z) was performed. *p* values were calculated according to the statistical test run. Effect sizes are calculated as Cohen’s *d* when a *t*-test was performed and *r* if a Wilcoxon signed-rank test was performed. Effect Size interpretation: Cohen’s *d*—large ≥ 0.80, moderate 0.50–0.79, small 0.21–0.49, negligible ≤ 0.20; *r*—large ≥ 0.50, moderate 0.30–0.49, small < 0.30. “% Responders” represents the percentage of participants in the sample who met the MCID for improvement.

**Table 8:** Reduction of impairment in Home (Parent) Settings.

	PARENT N = 38								
Scale	Mean-Pre (SD)	Mean-Post (SD)	Mean Difference (SD)	95% CI (L|U)	Test Statistic (T/Z)	*p* Value	Effect Size	95% CI of ES (L|U)	% Responders
IRS									
Relationship with Peers	3.5 (1.9)	2.53 (2.0)	0.97 (2.2)	0.19|1.7	2.516 (T)	0.017	0.43	0.08|0.78	
Relationship with Siblings	3.5 (1.9)	3.13 (1.8)	0.37 (1.9)	−0.31|1.1	−0.735 (Z)	0.46	−0.13	0.01|0.46	
Relationship with Parent	3.84 (1.8)	2.95 (1.8)	0.89 (1.7) **	0.34|1.4	−2.888 (Z)	0.004	−0.47	0.21|0.71	
Academic Progress	4.63 (1.4)	3.79 (1.7)	0.84(1.4) **	0.37|1.3	−3.316 (Z)	<0.001	−0.54	0.29|0.75	
Family Impact	3.55 (1.9)	2.76 (1.7)	0.79 (1.6) **	0.27|1.3	−2.974 (Z)	0.003	−0.48	0.19|0.68	
Self-esteem	3.84 (1.8)	3.08 (1.9)	0.76(1.7)	0.21|1.3	−2.625 (Z)	0.009	−0.42	0.15|0.66	
Overall	4.63 (1.0)	3.5 (1.7)	1.13(1.5) **	0.62|1.6	−3.826 (Z)	<0.001	−0.62	0.39|0.78	
Total	3.78 (1.1)	3.04 (1.5)	0.74 (1.1) **	0.36|1.1	3.939 (T)	<0.001	0.64	0.29|0.96	37%

** Indicates statistical significance after a Bonferroni correction of 0.05/8 = 0.006. For each variable, the normality assumption for *t*-tests was verified using a Shapiro–Wilks test. If the Shapiro–Wilks test indicated that the distribution of scores did not meet normality, a Wilcoxon signed-rank test was performed instead. In the “Test Statistic” column, this is indicated by a (T) or (Z) after the test statistic, indicating if a *t*-test (T) or a Wilcoxon test (Z) was performed. *p* values were calculated according to the statistical test run. Effect sizes are calculated as Cohen’s *d* when a *t*-test was performed and *r* if a Wilcoxon signed-rank test was performed. Effect Size interpretation: Cohen’s *d*—large ≥ 0.80, moderate 0.50–0.79, small 0.21–0.49, negligible ≤ 0.20; *r*—large ≥ 0.50, moderate 0.30–0.49, small < 0.30. “% Responders” represents the percentage of participants in the sample who met the MCID for improvement.

**Table 9:** Reduction of impairment in School (Teacher) Settings.

Scale	Mean-Pre (SD)	Mean-Post (SD)	Mean Difference (SD)	95% CI (L|U)	Test Statistic (T/Z)	*p* Value	Effect Size	95% CI of ES (L|U)	% Responders
IRS									
Relationship with Peers	2.5 (1.8)	1.69 (1.7)	0.81 (1.8)	0.08|1.5	−2.262 (Z)	0.024	−0.44	−0.63|−0.03	
Relationship with Teacher	2.81 (2.3)	1.69 (1.8)	1.12 (2.0)	0.32|1.9	2.892 (T)	0.008 **	0.57	0.15|0.98	
Academic Progress	3.96 (2.1)	3.46 (2.1)	0.50 (1.6)	−0.14|1.1	−1.584 (Z)	0.113	−0.31	−0.61|−0.01	
Classroom Impact	3 (2.3)	2.31 (2.3)	0.69 (2.1)	−0.14|1.5	−1.752 (Z)	0.08	−0.34	−0.69|−0.03	
Self-esteem	2.23 (2.3)	1.65 (1.9)	0.58 (2.5)	−0.43|1.6	1.176 (T)	0.251	0.23	−0.16|0.62	
Total	2.9 (1.7)	2.16 (1.5)	0.74 (1.4)	0.18|1.3	2.716 (T)	0.012	0.53	0.12|0.94	35%

** Indicates statistical significance after a Bonferroni correction of 0.05/6 = 0.008. For each variable, the normality assumption for *t*-tests was verified using a Shapiro–Wilks test. If the Shapiro–Wilks test indicated that the distribution of scores did not meet normality, a Wilcoxon signed-rank test was performed instead. In the “Test Statistic” column, this is indicated by a (T) or (Z) after the test statistic, indicating if a *t*-test (T) or a Wilcoxon test (Z) was performed. *p* values were calculated according to the statistical test run. Effect sizes are calculated as Cohen’s *d* when a *t*-test was performed and *r* if a Wilcoxon signed-rank test was performed. Effect Size interpretation: Cohen’s *d*—large ≥ 0.80, moderate 0.50–0.79, small 0.21–0.49, negligible ≤ 0.20; *r*—large ≥ 0.50, moderate 0.30–0.49, small < 0.30. “% Responders” represents the percentage of participants in the sample who met the MCID for improvement.

**Table 10:** Improvement in Domains of Functioning Based on Parent Ratings.

	PARENT N = 38								
Scale	Mean-Pre (SD)	Mean-Post (SD)	Mean Difference (SD)	95% CI (L|U)	Test Statistic (T/Z)	*p* Value	Effect Size	95% CI of ES (L|U)	% Responders
WFIRS—P									
Family	1.05 (0.74)	0.88 (0.73)	0.17 (0.46)	0.01|0.33	2.177 (T)	0.037	0.37	0.02|0.72	
School Learning	1.83 (0.71)	1.38 (0.85)	0.45 (0.67) **	0.22|0.67	−3.469 (Z)	<0.001	−0.58	0.32|0.78	
School Behavior	0.4 (0.41)	0.25 (0.27)	0.15 (0.30) **	0.04|0.25	−2.652 (Z)	0.008	−0.44	0.10|0.64	
Life Skills	1.2 (0.46)	0.94 (0.44)	0.26 (0.42) **	0.12|0.40	3.729 (T)	<0.001	0.61	0.26|0.96	
Self-Concept	0.95 (0.66)	0.86 (0.57)	0.09 (0.46)	−0.06|0.24	1.168 (T)	0.25	0.19	−0.13|0.52	
Social Activities	0.7 (0.58)	0.58 (0.55)	0.12 (0.29)	0.02|0.22	2.409 (T)	0.022	0.41	0.06|0.75	
Risky Activities	0.33 (0.24)	0.3 (0.25)	0.03 (0.19)	−0.03|0.10	1.087 (T)	0.285	0.19	−0.16|0.53	
Total	0.89 (0.36)	0.7 (0.39)	0.19 (0.28) **	0.09|0.28	4.075 (T)	<0.001	0.66	0.31|1.0	37%

** Indicates statistical significance after a Bonferroni correction 0.05/8 = 0.006. For each variable, the normality assumption for *t*-tests was verified using a Shapiro–Wilks test. If the Shapiro–Wilks test indicated that the distribution of scores did not meet normality, a Wilcoxon signed-rank test was performed instead. In the “Test Statistic” column, this is indicated by a (T) or (Z) after the test statistic, indicating if a *t*-test (T) or a Wilcoxon test (Z) was performed. *p* values were calculated according to the statistical test run. Effect sizes are calculated as Cohen’s *d* when a *t*-test was performed and *r* if a Wilcoxon signed-rank test was performed. Effect Size interpretation: Cohen’s *d*—large ≥ 0.80, moderate 0.50–0.79, small 0.21–0.49, negligible ≤ 0.20; *r*—large ≥ 0.50, moderate 0.30–0.49, small < 0.30. “% Responders” represents the percentage of participants in the sample who met the MCID for improvement.

**Table 11:** Improvement in Academic Performance.

	TEACHER N = 26							
Scale	Mean-Pre (SD)	Mean-Post (SD)	Mean Difference (SD)	95% CI (L|U)	Test Statistic (T)	*p* Value	Effect Size	95% CI of ES (L|U)
APRS								
Academic Success	20.88 (5.6)	22.28 (5.6)	−1.4 (3.27)	−2.75|−0.05	−2.13	0.043	−0.43	−0.83 |−0.01
Impulse Control	8.2 (1.8)	8.96 (1.7)	−0.76 (1.66)	−1.45|−0.07	−2.28	0.032	−0.46	−0.86|−0.04
Academic Productivity	34.33 (10.1)	38.5 (10.1)	−4.17 (7.06) **	−7.15|−1.18	−2.89	0.008	−0.59	−1.02|−0.15
Total Score	54.08 (13.5)	59.31 (13.6)	−5.23 (9.01) **	−8.87|−1.59	−2.96	0.007	−0.58	−0.99|−0.16

** Indicates statistical significance after a Bonferroni correction of 0.05/4 = 0.012. Effect size (Cohen’s *d*) interpretation: large ≥ 0.80, moderate 0.50–0.79, small 0.21–0.49, negligible ≤ 0.20.

## Data Availability

The data supporting the conclusions of this article are the property of Revibe Technologies and therefore not publicly available. The authors agree to share the de-identified data, without undue reservation.
